# Age differences in diffusivity in the locus coeruleus and its ascending noradrenergic tract

**DOI:** 10.1016/j.neuroimage.2022.119022

**Published:** 2022-02-19

**Authors:** Shai Porat, Francesca Sibilia, Josephine Yoon, Yonggang Shi, Martin J. Dahl, Markus Werkle-Bergner, Sandra Düzel, Nils Bodammer, Ulman Lindenberger, Simone Kühn, Mara Mather

**Affiliations:** a University of Southern California, Dept. of Gerontology, United States; b University of Southern California, Keck School of Medicine, United States; c Max Planck Institute for Human Development, Center for Lifespan Psychology, Germany

**Keywords:** Locus coeruleus, Noradrenergic bundle, Diffusion, Neuroimaging, Aging

## Abstract

The noradrenergic locus coeruleus (LC) is a small brainstem nucleus that promotes arousal and attention. Recent studies have examined the microstructural properties of the LC using diffusion-weighted magnetic resonance imaging and found unexpected age-related differences in fractional anisotropy - a measure of white matter integrity. Here, we used two datasets (Berlin Aging Study-II, *N* = 301, the Leipzig Study for Mind-Body-Emotion Interactions, *N* = 220), to replicate published findings and expand them by investigating diffusivity in the LC’s ascending noradrenergic bundle. In younger adults, LC fractional anisotropy was significantly lower, compared to older adults. However, in the LC’s ascending noradrenergic bundle, we observed significantly higher fractional anisotropy in younger adults, relative to older adults. These findings indicate that diffusivity in the LC versus the ascending noradrenergic bundle are both susceptible to structural changes in aging that have opposing effects on fractional anisotropy.

## 1. Introduction

The locus coeruleus (LC) is the brain’s primary source for noradrenaline (F. S. Giorgi et al., 2020a; Khanday et al., 2016; Lee et al., 2018), influencing arousal and attention (Aston-Jones and Waterhouse, 2016; Dahl et al., 2020; Mather, 2020; Mather and Harley, 2016; McGregor and Siegel, 2010; Sara, 2009). The LC also has widespread cortical projections that are susceptible to neurodegeneration (Aston-Jones and Waterhouse, 2016; Loizou, 1969; Loughlin et al., 1982; Morris et al., 2020). Notably, the human LC is the primary site of early abnormal tau pathology (Braak and Del Trecidi, 2015; Liu et al., 2020; Mather and Harley, 2016) and until recently, *in vivo* microstructural properties of the LC were mostly unexplored (Edlow et al., 2016, 2012; Langley et al., 2020).

Recently, Langley et al. (2020) examined the diffusive properties of the LC using diffusion-weighted MRI. They observed *higher* fractional anisotropy in the LC of older adults, compared with younger adults. Fractional anisotropy is widely used as a measure of structural integrity (higher fractional anisotropy typically indicates healthier axons) and has a strong inverse correlation with mean or radial diffusivity (Beck et al., 2021; Bhagat and Beaulieu, 2004; Kantarci et al., 2017; Kiely et al., 2022; Kochunov et al., 2012). With aging, older adults typically display lower fractional anisotropy and higher mean diffusivity in white and gray matter compared with younger adults (Beck et al., 2021; Kantarci, 2014; Lawrence et al., 2021; Medina and Gaviria, 2008b; Rose et al., 2008; Sullivan and Pfefferbaum, 2006; A. N. Voineskos et al., 2012a). In addition, gray matter also typically shows lower fractional anisotropy and higher mean diffusivity in Alzheimer’s disease (Kantarci, 2014; Rose et al., 2008; Weston et al., 2015). Thus, Langley’s findings are the opposite of typical white matter age effects.

Given the surprising nature of the increased fractional anisotropy seen in older adults’ LC compared with younger adults’ LC, we were interested in testing whether these age differences replicate in larger samples. Using two large datasets (Berlin Aging Study-II, *N* = 301, (Delius et al., 2015), and the Leipzig Study for Mind-Body-Emotion Interactions, *N* = 220, (Babayan et al., 2019) of healthy young and older adults, we examined whether we could replicate LC fractional anisotropy findings as reported by Langley, et al.. We also compared fractional anisotropy in the LC with fractional anisotropy in the ascending noradrenergic bundle, which originates in the LC. To characterize diffusion properties within the ascending noradrenergic bundle, we relied on a probabilistic atlas of bilateral ascending noradrenergic fiber bundles originating in the LC and terminating in the transentorhinal cortex based on data from the Human Connectome Project (Sun et al., 2020; Tang et al., 2018).

## 2. Methods

Demographics and MRI sequence information across all datasets can be found in Tables 1 through Table 4. The first dataset we examined is the Berlin Aging Study II (BASE-II) (Bertram et al., 2014; Delius et al., 2015) from timepoint two. BASE-II information can be found online (https://www.base2.mpg.de/en). BASE-II participants signed written informed consent and received monetary compensation for participation. MRI acquisitions were approved by the ethics committees of the German Psychological Society (Delius et al., 2015). All experiments were performed in accordance with relevant guidelines and regulations. In short, BASE-II is a multi-disciplinary and multi-institutional longitudinal study sampling from Berlin’s population. Because the BASE-II study included LC-MRI contrast measures, we asked whether these measures were associated with measures of LC and noradrenergic bundle diffusivity. The LC-MRI index potentially reflects neuromelanin accumulation as a byproduct of NE synthesis. Hence, it is supposed to indicate functional NE-density within the LC. If a lower LC-MRI contrast indeed reflects impaired functionality of the LC–NE system, detrimental down-stream effects on pathways connecting the LC to the entorhinal cortex might be expected. Thus, we expect lower LC-MRI contrast ratios to be associated with lower diffusivity. BASE-II LC-MRI contrast values were previously quantified in a different study (Dahl et al., 2019). Briefly, participants completed two LC-sensitive brainstem scans (Table 2). To improve signal-to-noise ratio, scans were motion corrected and ratios were extracted from both scans, then averaged across subjects (Dahl et al., 2019). LC location was identified semi-automatically and each participant’s LC intensity values were extracted and averaged to obtain a reliable estimate (Dahl et al., 2019).

The second dataset we examined is the Leipzig Study for Mind-Body-Emotion Interactions (LEMON), for which extensive details can be found elsewhere (Babayan et al., 2019). The Declaration of Helsinki was followed in this study and the ethics committee at the medical faculty of the University of Leipzig (reference number 154/13-ff) approved the LEMON study protocol (Babayan et al., 2019). This cross-sectional study contains both young and older adults from Leipzig, Germany, and the surrounding area. Subject demographics in each study, with final N’s per dataset, are displayed in Table 1. We excluded subjects with poor quality diffusion-weighted scans, poor atlas registration, or missing data in statistical analyses. Poor scan and registration quality was determined through visual inspection Tables 2–4. contain LC-MRI contrast sequence parameters, structural MRI parameters, and diffusion-weighted MRI parameters across studies, respectively.

### 2.1. DWI processing

**Fig. 1.**
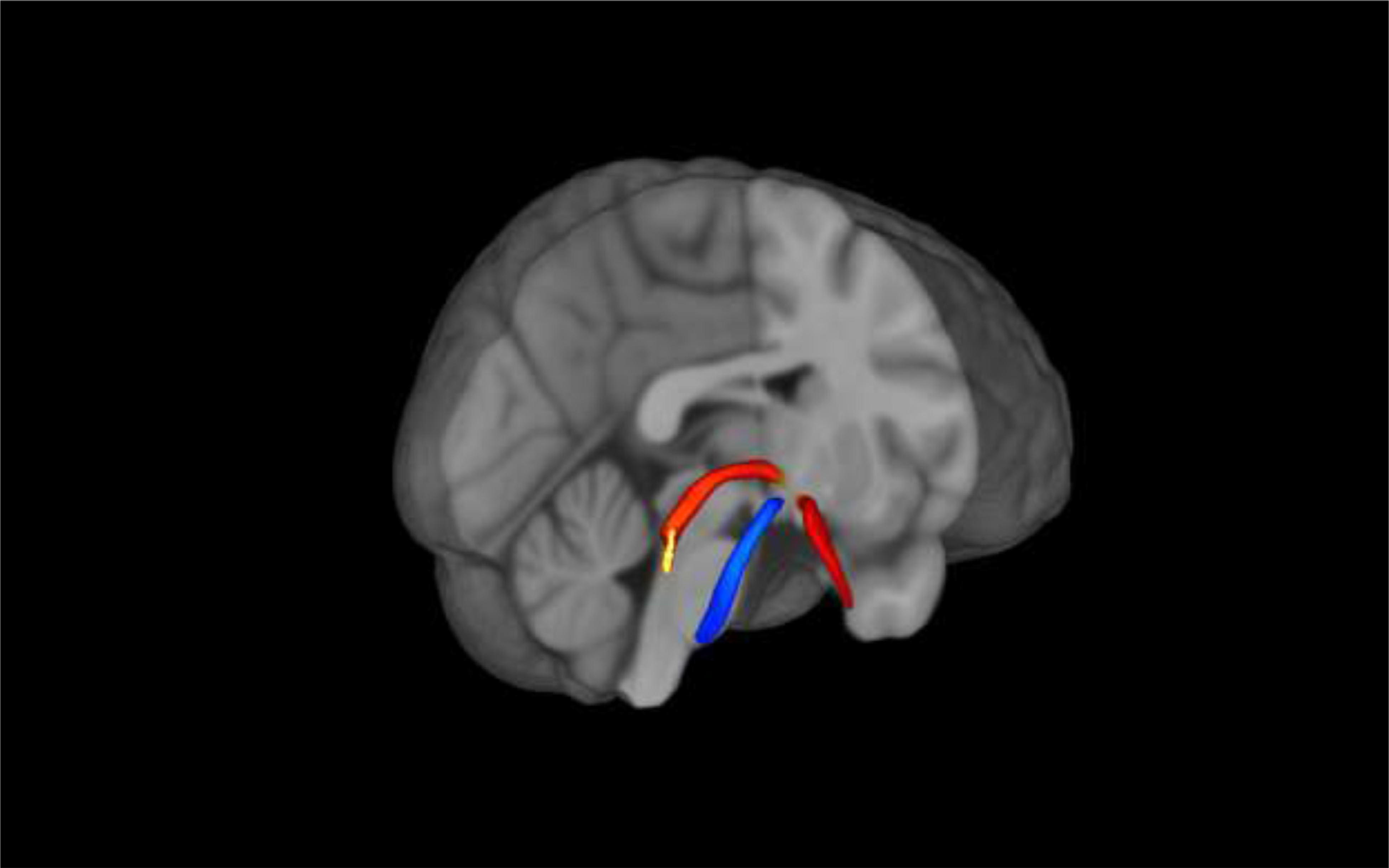
The ROI atlases of the locus coeruleus, noradrenergic bundle, and frontopontine tract. Note Fig. 1. displays the locus coeruleus (yellow), noradrenergic bundle (red), and frontopontine (blue) tracts registered to MNI152 space. The noradrenergic bundle is one continuous bundle (part of the temporal lobe segment is not pictured). (For interpretation of the references to color in this figure legend, the reader is referred to the web version of this article.)

Using University of Southern California’s Laboratory of Neuroimaging (LONI) Pipeline, we applied FSL’s *(v6.3)* eddy-current and motion correction, brain extraction tool, and resampling to isotropic resolution of 2mm^3^ (Dinov et al., 2009; Smith et al., 2004). We used MR-trix (*v3.1*) to compute fractional anisotropy (FA) and eigenvalue images (Tournier et al., 2019). With diffusion images, tensors are estimated for each participant and a whole brain fractional anisotropy image is created. Our atlas of the right and left LC nuclei was obtained from a LC meta-mask (Dahl et al., 2022) and the right and left noradrenergic bundles from Tang et al. (2020). As control regions, we utilized the previously defined right and left frontopontine tracts (Tang et al., 2018), which run along the ventral portion of the pons on either side of the basilar sulcus, terminating at the pontine nuclei Fig. 1. displays all three ROIs in MNI152 linear, 1 mm resolution space.

Both fractional anisotropy and atlas images were registered into MNI152 linear, 1 mm brain space. Using ANTS nonlinear registration (Avants et al., 2008; Sun et al., 2020) the atlases were then backwarped into individual subject space with nearest neighbor interpolation. Registration quality was visualized using an in-house MATLAB script (*MATLAB ver. R2019a*). After accurate atlas registration to individual subject space was confirmed with visual inspection, mean and radial diffusion images were created from eigen value images in MATLAB with custom scripts. Atlases were then converted into a binary mask and multiplied by the diffusion image to provide fractional anisotropy, mean, and radial diffusivity values along the atlases, per voxel, within the native space. Diffusivity values were then averaged to provide one diffusivity value per participant within each ROI.

Since the noradrenergic bundle overlaps with a portion of the LC atlas, we conducted an along-tract analysis of fractional anisotropy of the noradrenergic bundle. 50 equidistant points were imposed along the noradrenergic bundle as discussed elsewhere (Sun et al., 2020). Each point was averaged across participants within younger or older adult groups. Though the 50 equidistant points do not represent distinct anatomical regions, based on subject registrations, we can approximate the first 10 points to represent most of the LC and points 30–50 represent areas of the entorhinal cortex. Fractional anisotropy along the tract, is also represented visually on the Y-axis, against each of the 50 equidistant points between younger and older adults, shown in Figs. 4 and 5.

### 2.2. Statistical analyses

All statistical analyses were conducted using the R software (Team, 2020) with tidyverse and various additional packages (Ahlmann-Eltze, 2019; Aust and Barth, 2020; Kassambara; Lenth, 2021; Sjoberg et al., 2021; Wicham, 2017; Wickham, 2016; Xie, 2021). Correlation coefficients and 95% confidence intervals were used to identify the relationship between LC-MRI contrast and diffusivity measurements. Diffusivity and fractional anisotropy, mean diffusivity, and radial diffusivity values in the LC, ascending noradrenergic bundle, and frontopontine tract were analyzed within each dataset using a 2 × 2 × 3 × 2 factorial design in which age (younger, older) and gender (female, male) were between-subject factors and ROI (noradrenergic bundle, locus coeruleus, frontopontine tract) and hemisphere (left, right) were repeated-measures factors.

To clarify the significant interactions of age and the 3-level ROI factors, we conducted two follow-up ANOVAs with the ROI factor reduced to 2 levels to separately contrast the control frontopontine tract with each of the other two ROIs (figure and table in supplementary material). Levene’s tests were used to explore ANOVA assumptions of equal variances. We report effect sizes using ${\hat{\eta }}_{G}^{2}\;$ (generalized eta squared) for ANOVA effects and provide 95% confidence intervals to allow for comparisons across means. Greenhouse-Geisser correction was automatically computed for ANOVA departures from sphericity. For the along-tract analyses, *t*-tests were conducted for fractional anisotropy at each of the 50 equidistant points between younger and older adults. *P* values were false-discovery rate adjusted and surviving points of significant FA differences between age groups are plotted in Figs. 4 and 5. Our focus was on fractional anisotropy, but we include mean and radial diffusivity findings in the supplementary material. Lastly, to investigate if LC-FA diffusivity is associated with noradrenergic bundle-FA diffusivity, we conducted Pearson correlations and t-tests for each dataset.

## 3. Results

### 3.1. LC-MRI contrasts and DTI in BASE-II

In the BASE-II dataset, there were no significant differences between young and older adults’ overall LC-MRI contrast values (Bachman et al., 2021; Dahl et al., 2019). We also did not observe significant associations between LC-MRI contrast and diffusivity in either the LC or ascending noradrenergic bundle. Correlation coefficients with FA and 95% confidence intervals for younger and older adults are displayed in Table 5 and Table 6, respectively. Previous studies have observed no overall age differences, but spatially confined age differences between caudal and rostral regions of the LC have been observed with LC-MRI contrast (Bachman et al., 2021; Dahl et al., 2019).

### 3.2. Fractional anisotropy in the LC is higher in older adults, relative to younger adults

Complete ANOVA tables for fractional anisotropy across datasets are displayed in Tables 7–9. Here in the text, we report the significant ANOVA interactions involving Age and ROI. In the BASE-II and LEMON datasets, we observed a significant interaction of Age x ROI for fractional anisotropy, *F*(1.57, 468.27) = 27.18, *p* < .001, ${\overset{ˆ}{\eta }}_{G}^{2}\;=\;0.033\;$, and *F*(1.79, 386.34) = 26.07, *p* < .001, ${\overset{ˆ}{\eta }}_{G}^{2}\;=\;0.035\;$, respectively (Table 7 and Table 8). We also observed a significant 3-way interaction of Age × ROI × Hemisphere for fractional anisotropy, *F*(1.62, 483.01) = 6.49, *p* = .003, ${\overset{ˆ}{\eta }}_{G}^{2}\;=\;0.05\;$, and *F*(1.63, 352.15) = 5.50, *p* = .008, ${\overset{ˆ}{\eta }}_{G}^{2}\;=\;0.04\;$ = 0.04, in the BASE-II and LEMON datasets, respectively.

**Fig. 2.**
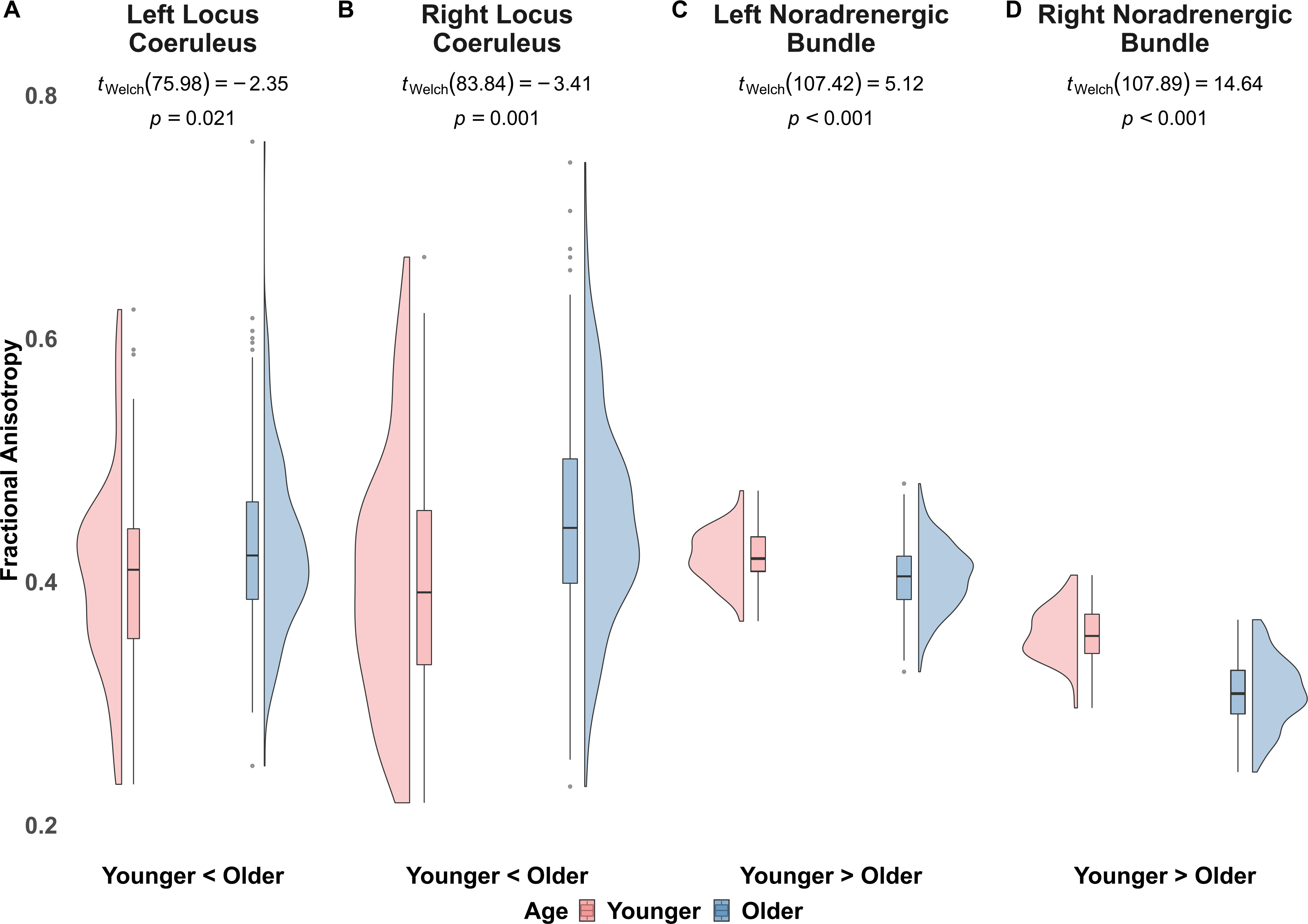
BASE-II fractional anisotropy in left and right locus coeruleus and noradrenergic bundles in younger and older adults. Note Fig. 2. displays fractional anisotropy between younger and older adults from the BASE-II cohort. In the left locus coeruleus (A) and right locus coeruleus (B), we observed lower fractional anisotropy in younger adults, compared to older adults. In the left noradrenergic bundle (C) and right noradrenergic bundle (D)we observed higher fractional anisotropy in younger adults, relative to older adults.

**Fig. 3.**
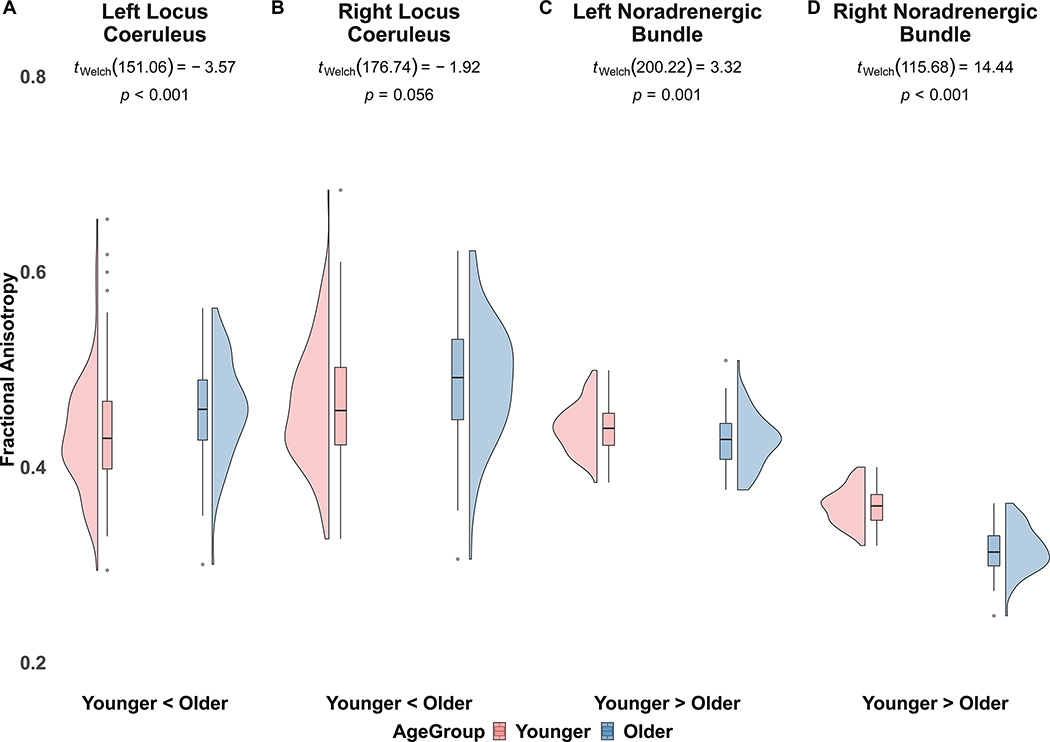
LEMON fractional anisotropy in left and right locus coeruleus and noradrenergic bundles in younger and older adults. Note Fig. 3. displays our BASE-II replicated fractional anisotropy findings between younger and older adults in the LEMON dataset. In the left locus coeruleus (A) and right locus coeruleus (B), we observed lower fractional anisotropy in younger adults, compared to older adults. In the left noradrenergic bundle (C) and right noradrenergic bundle (D) we observed higher fractional anisotropy in younger adults, relative to older adults.

Tables 9 and 10 report means and 95% confidence intervals for fractional anisotropy for each ROI between age groups, in each hemisphere. We observed significantly less fractional anisotropy in the LC and significantly more fractional anisotropy in the noradrenergic bundle of younger adults compared to older adults, in both the BASE-II and LEMON datasets (Tables 9 and 10; Figs. 2 and 3). We observed no significant differences in frontopontine tract fractional anisotropy between younger and older adults in either BASE-II or LEMON datasets.

Separate ANOVAs with the “ROI” factor either contrasting the frontopontine tract and noradrenergic bundle fractional anisotropy or contrasting the frontopontine tract and LC fractional anisotropy both yielded significant Age X ROI effects (Table 9; Figs. 1–4 in supplementary material), *F*(299) = 26.16, *p* < .001, ${\hat{\eta }}_{G}^{2}\;=\;0.024\;$, and *F*(299) = 9.72, *p* = .002, ${\hat{\eta }}_{G}^{2}\;=\;0.009\;$, respectively in BASE-II. As well as in LEMON datasets, *F*(216) = 24.34, *p* < .001, ${\overset{ˆ}{\eta }}_{G}^{2}\;=\;0.025$, and *F*(216) = 5.23, *p* = .023, ${\hat{\eta }}_{G}^{2}\;=\;0.006\;$, respectively. This indicates that the effects of age on fractional anisotropy in the LC and noradrenergic bundle each differed significantly from the control region, in opposite directions.

**Fig. 4.**
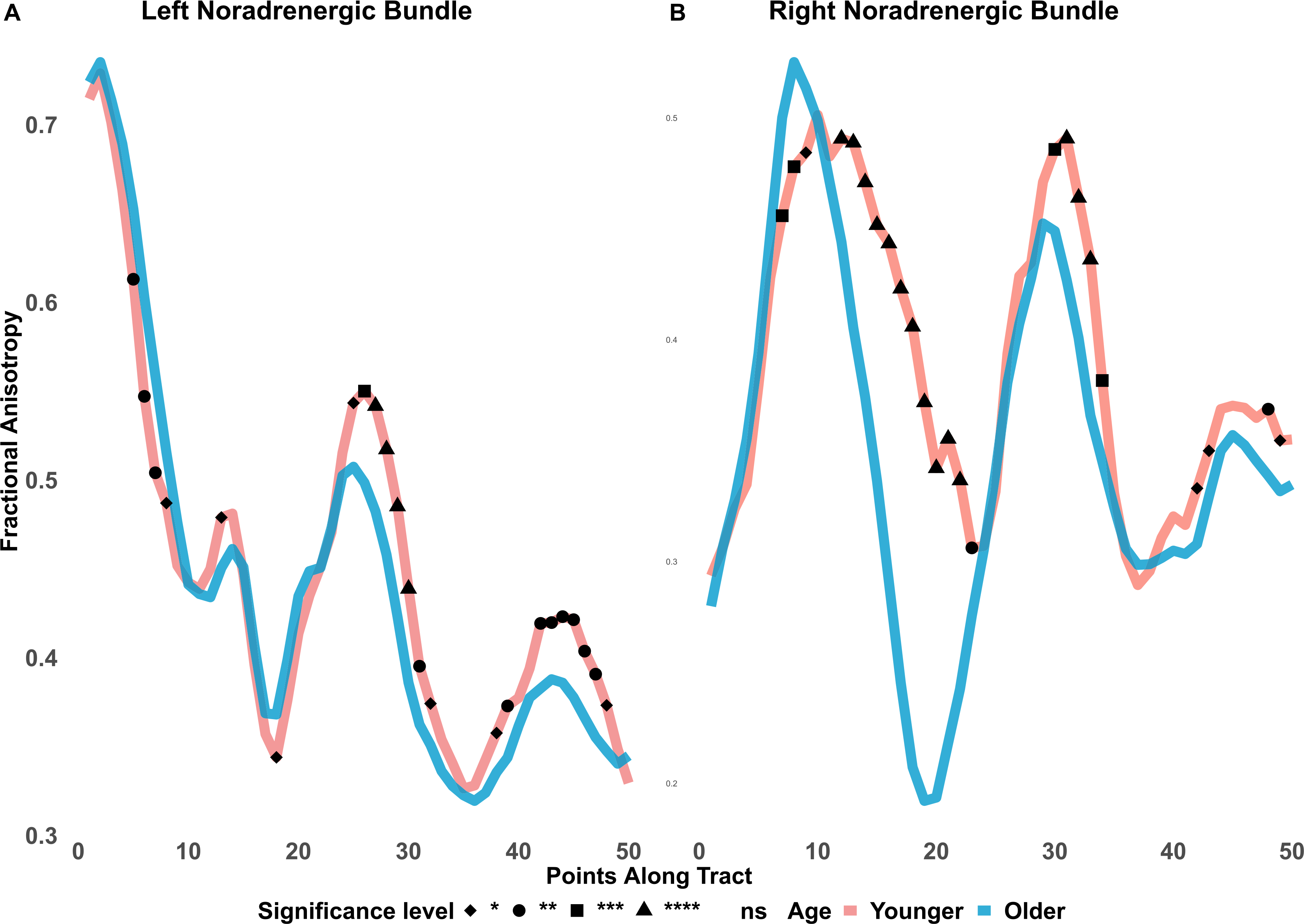
BASE-II fractional anisotropy along the noradrenergic bundle. Note. ns = not significant; ns not assigned shape. Fractional anisotropy differences between younger and older adults are shown along the noradrenergic bundle. The bundle was divided into 50 equidistant points and mean fractional anisotropy was calculated for each age group at each point. Younger adults had significantly lower fractional anisotropy in the first 10 points of the noradrenergic bundles which would correspond to the area of the locus coeruleus. In contrast, around the entorhinal cortex, younger adults show higher fractional anisotropy, compared to older adults. * *p* ≤ 0.05. * * *p* ≤ 0.01. * * * *p* ≤ 0.001. * * * * *p* ≤ 0.0001. FDR adjusted.

**Fig. 5.**
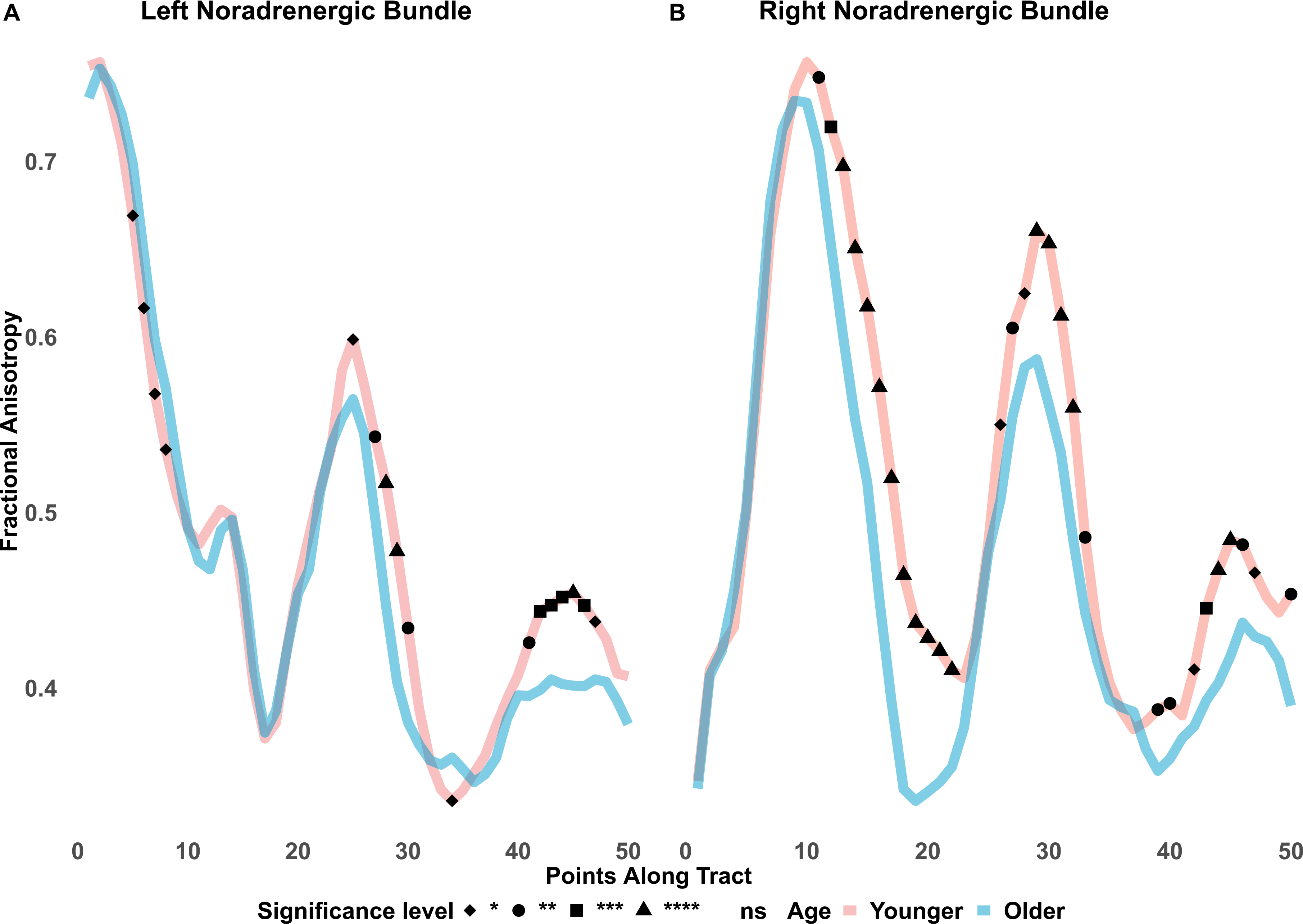
LEMON fractional anisotropy along the noradrenergic bundle. Note, ns = not significant; ns not assigned shape. Fractional anisotropy differences between LEMON younger and older adults are shown along the noradrenergic bundle. The bundle was divided into 50 equidistant points and mean fractional anisotropy was calculated for each age group at each point. Younger adults had significantly lower fractional anisotropy in the first 10 points of the noradrenergic bundle, more so in the left than right, which would correspond to the area of the locus coeruleus. In contrast, younger adults showed higher fractional anisotropy and significantly greater differences around the entorhinal cortex, compared to older adults. * *p* ≤ 0.05. ** *p* ≤ 0.01. *** *p* ≤ 0.001. **** *p* ≤ 0.0001. FDR adjusted.

The BASE-II and LEMON along-tract analyses (Figs. 4 and 5) show effects that are consistent with the LC and noradrenergic bundle results described above. Along the first 10 points, which approximately represent regions close to the LC, younger adults display significantly lower fractional anisotropy, relative to older adults. In the remaining tract points, younger adults had higher fractional anisotropy, relative to older adults, with significant age differences toward the end of the tract, in the region of the entorhinal cortex.

Finally, we observed significant positive correlations between LC fractional anisotropy and noradrenergic bundle fractional anisotropy within the BASE-II older adult cohort in the left and right hemispheres, *r*(243) = 0.24, *p* < .001 and *r*(243) = 0.19, *p* < .001, respectively. However, only three percent of the variance was accounted for (R^2^_adj_ = 0.03). We did not observe any significant relationship in the BASE-II young adult cohort. We were also not able to replicate these findings in the LEMON dataset.

## 4. Discussion

Unmyelinated neurons and numerous innervations to blood capillaries may expose the LC to toxins throughout aging (Bekar et al., 2012; Giorgi et al., 2020a; Raichle et al., 1975). During the waking day, the LC has a high constant spiking rate which accumulates oxidative stress in the mitochondria of LC neurons (Weinshenker, 2018). In addition, excess noradrenaline not repackaged into synaptic vesicles promotes LC tau pathology (Kang et al., 2020). Existing evidence also suggests that older adults may be more at risk of these negative side effects of LC activity due to higher tonic activity levels (Gutchess et al., 2020; Mather, 2021; Weinshenker, 2018).

Fractional anisotropy has been observed to correlate with white matter integrity, increasing until the age of about 35–40 and decreasing into late life or with disease (Beck et al., 2021; Kiely et al., 2022; Kochunov et al., 2012; Li et al., 2016). Additionally, mean, and radial diffusivity are typically negatively correlated with fractional anisotropy (Beaudet et al., 2020; Beck et al., 2021; Kiely et al., 2022; Li et al., 2016). Here, using two publicly available datasets (Babayan et al., 2019; Delius et al., 2015), we examined the age-related diffusivity of the LC, ascending noradrenergic bundle, and, as a control, frontopontine white matter tracts. We replicated Langley et al. (2020) findings of higher fractional anisotropy in the LC in older adults compared with younger adults, across two large datasets (BASE-II; LEMON).

While fractional anisotropy tended to be higher in older than younger adults within the LC itself, older adults typically showed lower fractional anisotropy than younger adults along the noradrenergic bundle white-matter ascending tract, a typical age-related pattern in white matter (Beck et al., 2021; Medina and Gaviria, 2008; Sibilia et al., 2017; Sullivan and Pfefferbaum, 2006; Voineskos et al., 2012a). The lack of associations observed in our datasets between LC fractional anisotropy and noradrenergic bundle fractional anisotropy may suggest these two regions are affected by aging independently.

In the BASE-II and LEMON datasets, age differences in the noradrenergic bundle contrasted with a lack of age differences in the right and left control white-matter frontopontine tracts, suggesting that the age effects in the noradrenergic ascending tract reflect more than just a global change in white matter. Thus, together, these data indicate that diffusivity properties of the LC and its ascending noradrenergic tract are affected by aging in opposite ways. Our findings of age differences in fractional anisotropy in the LC and its ascending tracts extend a growing set of observations of age differences in the structure of the LC in aging (Brickman et al., 2012; Chen et al., 2014; Chu et al., 2021; Dahl et al., 2021; Dahl et al., 2020; Langley et al., 2020; Morris et al., 2020; Sun et al., 2019).

To date, most in vivo findings of LC structure have relied on LC-MRI sequences that show a cross-sectional increase in LC-neuromelanin sensitive contrast from young adulthood until around age 57, at which point it levels off or declines (Liu et al., 2019), potentially suggesting a gradual accumulation of neuromelanin followed by noradrenergic degeneration. One of the two data sets we examined (BASE-II) included neuromelanin-sensitive scans. While LC-MRI contrast has been validated to correlate with the location of neuromelanin (Keren et al., 2015), it is not yet entirely clear what factors contribute to currently employed LC-MRI contrast measures. Current hypotheses include presence of macromolecules (Priovoulos et al., 2020), density of water protons (Watanabe et al., 2019), and the relative proportion of macromolecular protons to free water protons (Trujillo et al., 2019).

In the BASE-II dataset, there were no significant correlations between LC-MRI contrast from those scans and diffusivity measures from the LC or noradrenergic bundle. This suggests that the diffusivity differences reflect different structural changes than the LC-sensitive scans. An important future research objective should be to examine the relationship between LC diffusivity measures and cognition, or markers of brain health, as has been done for LC-MRI contrast (Clewett et al., 2016; Dahl et al., 2019; Langley et al., 2020). One initial study along these lines found that medial and radial diffusivity in the LC-thalamus tract was correlated with memory performance in an older cohort (Langley et al., 2021).

Our results raise the question of what properties of the LC are changing to lead its tissue to show higher fractional anisotropy with age. One possibility could be an increase in inflammation that restricts fluid flow, as animal research has demonstrated that increases in microglial density affect diffusivity, as measured using an orientation dispersion index (Yi et al., 2019). Another possibility is that the age differences in LC diffusivity relate to age differences in LC tonic activity levels. Although still an open question, various findings suggest that the LC is more tonically active in aging (Mather, 2020; Weinshenker, 2018). Age differences in tonic levels of LC could contribute to differences in diffusivity as neuronal activity increases neuronal volume, while shrinking the volume of the surrounding fluid-filled spaces (Abe et al., 2017; Iwasa et al., 1980; Le Bihan et al., 2006; Nunes et al., 2021; Svoboda and Syková, 1991; Tirosh and Nevo, 2013).

Mean and radial diffusivity in the LC also showed some age differences (results and tables provided in the supplementary material), although not as pronounced as fractional anisotropy. In the BASE-II dataset, mean diffusivity in the LC was significantly higher in younger adults, compared to older adults. In the LEMON dataset, mean diffusivity was significantly higher in the left LC of younger adults, compared to older adults. Though the cause for these laterality effects is not known, the BASE-II dataset is composed of mostly older adults, while the LEMON has more younger adults. Given the LC’s proximity to the fourth ventricle, older adults may be susceptible to neurodegeneration within the LC as well as partial volume effects (Langley et al., 2020; Liu et al., 2017; Sun et al., 2020).

Because the noradrenergic bundle overlaps with the LC atlas, we conducted an along-tract analysis for the noradrenergic bundle fractional anisotropy. As expected, we observed significantly lower FA in the first 10 points of the noradrenergic bundle, which anatomically approximately represent regions of the locus coeruleus, in younger adults compared with older adults. Changes in radial diffusivity along the noradrenergic bundle of cognitively impaired older adults from the Alzheimer’s Disease Neuroimaging Initiative were significantly greater, compared to healthy controls, around the area of the LC and again as the tract approached the hippocampus (Sun et al., 2020).

While most studies comparing diffusivity in younger and older adults focus on white matter, a growing number of studies have started to examine diffusivity differences in gray matter in cortical and subcortical nuclei. Patients with Alzheimer’s disease generally show less fractional anisotropy and greater mean diffusivity than age-matched healthy adults (Weston et al., 2015). However, studies following people with autosomal dominant familial Alzheimer’s disease have found increased mean diffusivity in gray matter regions during the pre-symptomatic period, and older adults with significant memory decline show lower diffusivity in the posterior cingulate/precuneus region (Jacobs et al., 2013). As Langley et al., suggested, age-related LC degeneration may result in restricted diffusion within older adults (Langley et al., 2020). Fractional anisotropy also shows a positive correlation with age in the caudate, putamen and globus pallidus in a healthy cohort aged 10–52 (Pal et al., 2011). Thus, the LC may not be the only brain region showing lower fractional anisotropy in older adults.

### 4.1. Limitations

Crossing fibers may indicate opposite or unexpected relationships with diffusivity values that may be related to our unexpected findings (Lee et al., 2015; Oouchi et al., 2007). Despite the limitations of DTI, it remains a valuable tool that may help us to better understand the LC *in-vivo* within humans. In general, our datasets were comprised of younger and older adults that had no neurological or known sleep disorders and may not reflect the general aging population. We also did not examine axial diffusivity. Due to partial volume constraints, the locus coeruleus ROI may be contaminated by white matter and CSF (given the position near the 4th ventricle). However, given the opposite findings in the ascending white matter tract, we were still able to extract meaningful signal.

## 5. Conclusions

In this study, we identified unique associations of LC diffusivity in the context of healthy adults across two different data sets. We consistently observed lower fractional anisotropy in the locus coeruleus of younger adults, compared to older adults but higher fractional anisotropy in the ascending noradrenergic bundle of younger adults, compared to older adults. Fractional anisotropy is a measurement of structural integrity, and these age findings add to a growing literature highlighting age-related differences involving the locus coeruleus. To our knowledge, this is the first study to compare diffusivity differences *in-vivo* in the locus coeruleus versus noradrenergic bundle (Table 3).

## Supplementary Material

1

## Figures and Tables

**Table 1:** Demographics for each dataset.

	Younger Adults	Older Adults	*p* ^a^

Berlin Aging Study-II (BASE-II)			
Age in Years ^b^	35.90 (3.67)	75 0.65 (4.05)	<0.001
Sex ^c^			0.6
Male	39 (67)	154 (63)	
Female	19 (33)	89 (37)	
Total	58	243	
Leipzig Study for Mind-Body-Emotion Interactions (LEMON)			
Age in Years	25.10 (3.10)	67.60 (4.70)	<0.001
Sex			0.004
Male	105 (70)	35 (49)	
Female	44 (30)	36 (51)	
Total	149	71	

a Statistical tests performed: chi-square test of independence (for comparisons across sexes and sleep deprivation conditions); Wilcoxon rank-sum test (for age).

b Statistics presented: Mean (SD).

c Statistics presented: n (% of total).

**Table 2:** LC-MRI contrast sequence parameters.

BASE-II	Item	Duration or Size

Scanner	3-Tesla Siemens Magnetom Tim Trio	
Head-coil	12-channel	
Sequence	High-resolution, two-dimensional T1-weighted turbo-spin echo (TSE) sequence aligned perpendicularly to the plane of the respective participant’s brainstem	2 * 5.9 min
Parameters	Repetition Time	600ms
	Echo Time	11ms
	Inversion Time	
	Flip Angle	120 °
	Bandwidth	287 Hz/pixel
	FOV	350mm
	Slice Thickness	2.5 mm isotropic

**Table 3:** Structural MRI sequence parameters in each study.

	Item	Duration or Size

Berlin Aging Study-II (BASE-II)		
Scanner	3-Tesla Siemens Magnetom Tim Trio	
Head coil	12-channel	
Sequence	T1-weighted magnetization prepared gradient-echo (MPRAGE)	9:2 min
Parameters	Repetition Time	2500ms
	Echo Time	4.77ms
	Inversion Time	1100ms
	Flip Angle	7 °
	Bandwidth	140 Hz/pixel
	FOV	256mm
	Slice Thickness	1 mm isotropic
Other	The LC-contrast and structural MRI scans were acquired when participants returned for BASE-II evaluations at Time 2. Pre-scan normalize, and 3D distortion correction options were enabled.	
Leipzig Study for Mind-Body-Emotion Interactions (LEMON)		
Scanner	3-Tesla Siemens Magnetom Verio	
Head coil	32-channel	
Sequence	Magnetization Prepared 2 Rapid Acquisition Gradient Echoes (MP2RAGE)	8:22 min
Parameters	Repetition Time	5000ms
	Echo Time	2.92ms
	Inversion Time 1/2	700/2500ms
	Flip Angle 1/2	4/5°
	Bandwidth	240 Hz/pixel
	FOV	256mm
	Slice Thickness	1 mm isotropic
Other	For more information please see Babayan et al. (2019)	

**Table 4:** Diffusion MRI sequence parameters in each study.

	Item	Duration or Size

Berlin Aging Study-II (BASE-II)		
Scanner	3-Tesla Siemens Magnetom Tim Trio	
Head coil	12-channel	
Sequence	Transverse plane and seven volumes collected without diffusion weighting (*b* = 0).	12:52 min
Parameters	Repetition Time	11000ms
	Echo Time	98ms
	Gradient Directions	60
	Diffusion Weighting	*b* = 1000 s/mm^2^
	Bandwidth	1628 Hz/pixel
	FOV	218mm
	Slice Thickness	1.7 mm isotropic
Other	Generalized auto-calibrating partially parallel acquisitions (GRAPPA) acceleration factor = 2 in single-shot, echo-planar imaging. For more details, see Dahl et al. (2019a) and Bender et al. (2019).	
Leipzig Study for Mind-Body-Emotion Interactions (LEMON)		
Scanner	3-Tesla Siemens Magnetom Verio	
Head coil	32-channel	
Sequence	Transverse plane and seven volumes collected without diffusion weighting (*b* = 0).	9:27 min
Parameters	Repetition Time	7000ms
	Echo Time	80ms
	Gradient Directions	60
	Diffusion Weighting	*b* = 1000 s/mm^2^
	Bandwidth	1502 Hz/pixel
	FOV	220mm
	Slice Thickness	1.7 mm isotropic
Other	For more information please see (Babayan et al., 2019)	

**Table 5:** Younger adults LC-MRI contrast correlations with confidence intervals.

Variable	LC-MRI Contrast

Noradrenergic bundle FA – Left hemisphere	−0.07 [−0.31, 0.18]
Noradrenergic bundle FA – Right hemisphere	−0.09 [−0.33, 0.16]
Locus Coeruleus FA – Left hemisphere	−0.02 [−0.27, 0.23]
Locus Coeruleus FA – Right hemisphere	−0.06 [−0.30, 0.19]
Frontopontine FA – Left hemisphere	.04 [−0.21, 0.29]
Frontopontine FA – Right hemisphere	.15 [−0.10, 0.39]

*Note.* Values in square brackets indicate the 95% confidence interval for each correlation.

**Table 6:** Older adults LC-MRI contrast correlations with confidence intervals.

Variable	LC-MRI Contrast

Noradrenergic bundle FA – Left hemisphere	−0.03 [−0.15, 0.10]
Noradrenergic bundle FA – Right hemisphere	−0.08 [−0.20, 0.05]
Locus Coeruleus FA – Left hemisphere	.05 [−0.08, 0.17]
Locus Coeruleus FA – Right hemisphere	−0.10 [−0.23, 0.02]
Frontopontine FA – Left hemisphere	−0.06 [−0.19, 0.06]
Frontopontine FA – Right hemisphere	−0.02 [−0.14, 0.11]

*Note.* Values in square brackets indicate the 95% confidence interval for each correlation.

**Table 7:** BASE-II fractional anisotropy mixed ANOVA.

Variable	*F*	${d\;f}_{1}^{GG}\;$	${d\;f}_{2}^{GG}\;$	*MSE*	*p*	${\hat{\eta }}_{G}^{2}\;$

Age (Younger, Older)	1.05	1	299	0.01	.306	.001
Gender (Female, Male)	7.33	1	299	0.01	.007	.007
ROI (LC, Noradrenergic Bundle, Frontopontine)	598.17	1.57	468.27	0.01	<0.001	.426
Hemisphere (Left, Right)	125.52	1	299	0.00	<0.001	.049
Age × Gender	1.21	1	299	0.01	.273	.001
Age × ROI	27.18	1.57	468.27	0.01	<0.001	.033
Gender × ROI	1.97	1.57	468.27	0.01	.151	.002
Age × Hemisphere	0.00	1	299	0.00	.980	.000
Gender × Hemisphere	0.17	1	299	0.00	.682	.000
ROI × Hemisphere	73.81	1.62	483.01	0.00	<0.001	.051
Age × Gender × ROI	0.46	1.57	468.27	0.01	.584	.001
Age × Gender × Hemisphere	0.46	1	299	0.00	.497	.000
Age × ROI × Hemisphere	6.49	1.62	483.01	0.00	.003	.005
Gender × ROI × Hemisphere	0.25	1.62	483.01	0.00	.729	.000
Age × Gender × ROI × Hemisphere	0.17	1.62	483.01	0.00	.802	.000

*Note.* “GG”: applies Greenhouse-Geisser correction to all within-subjects factors.

**Table 8:** LEMON fractional anisotropy mixed ANOVA.

Variable	*F*	${d\;f}_{1}^{GG}\;$	${d\;f}_{2}^{GG}\;$	*MSE*	*p*	${\hat{\eta }}_{G}^{2}\;$

Age (Younger, Older)	0.26	1	216	0.01	.608	.001
Gender (Female, Male)	0.72	1	216	0.01	.396	.002
ROI (LC, Noradrenergic Bundle, Frontopontine)	761.97	1.79	386.34	0.00	< 0.001	.513
Hemisphere (Left, Right)	412.73	1	216	0.00	< 0.001	.130
Age × Gender	0.38	1	216	0.01	.539	.001
Age × ROI	26.07	1.79	386.34	0.00	< 0.001	.035
Gender × ROI	2.22	1.79	386.34	0.00	.116	.003
Age × Hemisphere	9.17	1	216	0.00	.003	.003
Gender × Hemisphere	1.69	1	216	0.00	.195	.001
ROI × Hemisphere	308.54	1.63	352.15	0.00	< 0.001	.194
Age × Gender × ROI	0.57	1.79	386.34	0.00	.546	.001
Age × Gender × Hemisphere	4.70	1	216	0.00	.031	.002
Age × ROI × Hemisphere	5.50	1.63	352.15	0.00	.008	.004
Gender × ROI × Hemisphere	1.56	1.63	352.15	0.00	.214	.001
Age × Gender × ROI × Hemisphere	2.58	1.63	352.15	0.00	.088	.002

*Note.* “GG”: applies Greenhouse-Geisser correction to all within-subjects factors.

**Table 9:** BASE-II fractional anisotropy means, standard error, degrees of freedom and 95% confidence intervals.

BASE-II	Locus Coeruleus		Noradrenergic Bundle		Frontopontine Tract	
	Young Adult	Older Adult	Young Adult	Older Adult	Young Adult	Older Adult

Left Hemisphere						
*M* ^a^	0.396	0.426	0.421	0.403	0.563	0.567
*SE*	0.01	0.005	0.004	0.002	0.008	0.004
Lower CI	0.377	0.417	0.413	0.4	0.547	0.559
Upper CI	0.416	0.436	0.428	0.407	0.58	0.574
Right Hemisphere						
*M* ^a^	0.397	0.452	0.356	0.309	0.515	0.522
*SE*	0.014	0.007	0.004	0.002	0.008	0.004
Lower CI	0.369	0.439	0.348	0.306	0.5	0.515
Upper CI	0.424	0.465	0.363	0.313	0.53	0.529

*Note.* CI = confidence interval.

a degrees of freedom = 299.

**Table 10:** LEMON fractional anisotropy means, standard error, degrees of freedom and 95% confidence intervals.

LEMON	Locus Coeruleus		Noradrenergic Bundle		Frontopontine Tract	
	Young Adult	Older Adult	Young Adult	Older Adult	Young Adult	Older Adult

Left Hemisphere						
*M* ^a^	0.427	0.459	0.445	0.427	0.584	0.582
*SE*	0.005	0.006	0.003	0.005	0.006	0.008
Lower CI	0.418	0.446	0.438	0.418	0.572	0.567
Upper CI	0.437	0.472	0.451	0.436	0.595	0.597
Right Hemisphere						
*M* ^a^	0.473	0.487	0.365	0.315	0.497	0.503
*SE*	0.007	0.009	0.003	0.004	0.005	0.007
Lower CI	0.46	0.47	0.359	0.308	0.487	0.489
Upper CI	0.486	0.505	0.37	0.323	0.508	0.517

*Note*. CI = confidence interval.

a degrees of freedom = 216.
